# COX-2 and PPARγ expression are potential markers of recurrence risk in mammary duct carcinoma in-situ

**DOI:** 10.1186/1471-2407-8-36

**Published:** 2008-01-31

**Authors:** Swati Kulkarni, Deepa B Patil, Leslie K Diaz, Elizabeth L Wiley, Monica Morrow, Seema A Khan

**Affiliations:** 1 Lynn Sage Breast Center and Feinberg School of Medicine of Northwestern University, Chicago USA; 2 Department of Surgery Roswell Park Cancer Institute, Buffalo USA; 3 Department of Surgery, Fox Chase Cancer Center Philadelphia USA; 4 Department of Pathology Abbott Northwestern Hospital, Minneapolis USA; 5 Department of Pathology, University of Illinois Medical Center Chicago USA

## Abstract

**Background:**

In women with duct carcinoma in-situ (DCIS) receiving breast conservation therapy (BCT), in-breast recurrences are seen in approximately 10%, but cannot be accurately predicted using clinical and histological criteria. We performed a case-control study to identify protein markers of local recurrence risk in DCIS.

**Methods:**

Women treated for DCIS with BCT, who later developed in-breast recurrence (cases) were matched by age and year of treatment to women who remained free of recurrence (controls).

**Results:**

A total of 69 women were included in the study, 31 cases and 38 controls. Immunohistochemical evaluation of DCIS tissue arrays was performed for estrogen receptor, progesterone receptor, HER-2/neu, cyclin D1, p53, p21, cycloxygenase-2 (COX-2) and peroxisome proliferator activated receptor γ (PPARγ). Two markers were significantly different between cases and controls on univariate analysis: strong COX-2 expression was associated with increased risk of recurrence, with 67% vs. 24% positivity in cases and controls p = 0.006; and nuclear expression of PPARγ was associated with protection from recurrence with 4% vs. 27% positivity in cases and controls, p = 0.024. In a multivariate model which included size, grade, COX-2 and PPARγ positivity, we found COX-2 positivity to be a strong independent risk factor for recurrence (OR 7.90, 95% CI 1.72–36.23)., whereas size and grade were of borderline significance. PPARγ expression continued to demonstrate a protective trend, (OR 0.14, 95% CI 0.06–1.84).

**Conclusion:**

Our findings suggest that COX-2 and PPARγ should be investigated further as biologic markers to predict DCIS recurrence, particularly since they are also potential therapeutic targets.

## Background

With the widespread use of screening mammography, the incidence of ductal carcinoma in-situ (DCIS) has increased steadily[1], so that DCIS now accounts for up to 20% of newly diagnosed breast cancers[2]. Management of localized DCIS has evolved from total mastectomy which is associated with an less than 2% ipsilateral recurrence risk to breast conservation therapy (BCT) and endocrine therapy where ipsilateral recurrence rates vary from 10%–40% [3,4]. Because of the increasing number of women being diagnosed with DCIS, the identification of those patients at high risk for a recurrence (invasive and non-invasive) is of increasing importance. Morphological factors such as grade and the presence of comedo-necrosis have been used thus far to predict recurrence, but lack sensitivity and are subject to variability of interpretation[5].

A number of molecular markers have been identified in invasive breast cancers that have predictive as well as prognostic value. Well known markers include the estrogen receptor α (ER) and progesterone receptor (PR) which are associated with improved outcomes and are predictive of response to tamoxifen therapy. HER-2/neu is a tyrosine kinase receptor related to the epidermal growth factor receptor family. Overexpression of HER-2/neu in invasive carcinoma is correlated with decreased relapse-free and overall survival, and resistance to hormonal and cytotoxic therapy[6]. In DCIS, HER-2/neu has been linked to adverse clinicopathologic characteristics including higher histological grade, comedo-necrosis and younger age. Additionally, HER-2/neu is found more frequently in DCIS associated with invasive cancer [7-10]. Cyclin D1, p53, and p21, are all proteins involved in cell cycle regulation and have prognostic value in invasive breast cancer as well [11-14]. All of these markers have been identified in DCIS recently, but their biologic significance is not fully understood, and long-term data relating these markers to recurrence risk in DCIS are extremely scant[7,15-17]. Two novel molecular markers are cycloxygenase-2 (COX-2) and peroxisome proliferator activated-receptor gamma (PPARγ). COX-2 overexpression has been shown to be up-regulated in many neoplastic and pre-neoplastic lesions including invasive breast cancer and DCIS [18-20]. COX-2 is the inducible form of cyclooxygenase, the rate-limiting enzyme for prostaglandin synthesis. PPARγs appear to play a role in the regulation of many physiologic functions such as lipid metabolism, atherogenesis, inflammation and cellular differentiation[21,22]. PPARγ expression has been studied in invasive cancers but not in DCIS thus far.

We have conducted an exploratory study to identify those molecular markers associated with increased or decreased ipsilateral in-breast recurrence after diagnosis of DCIS, in a population with long term follow-up; we opted for a case-control design because of the relative rarity of DCIS recurrence.

## Methods

Following IRB approval of the study, women diagnosed with DCIS and treated with breast conservation, who subsequently developed ipsilateral recurrence of ductal carcinoma, either invasive or non-invasive, (cases) were identified from the Northwestern Memorial Hospital tumor registry and the Lynn Sage Breast Center database from 1992–2002. We have used the term "breast conserving therapy" to include all women treated without mastectomy; of these 60% also received breast radiotherapy, which was not a standard component of BCT for DCIS during the earlier part of the study. Similarly, tamoxifen therapy was introduced into the treatment of DCIS over this time period, and just 32% of our study subjects were treated with tamoxifen. Only women who had their original surgery at Northwestern Memorial Hospital were included, because the tissue blocks at primary diagnosis were needed to create tissue microarrays. The recurrences were in the same region of the breast as the original DCIS, and were characterized as "local recurrences" by the treating physicians. These cases were age-matched (within two years) to a set of control subjects diagnosed with DCIS and treated with breast conservation in the same calendar year, who did not recur over the same time period (controls). Matching for tumor size and grade was not possible because of the relative scarcity of younger women with DCIS and the limited number of women undergoing breast conservation early in study period. Clinical information recorded on chart review included age, race, age of menarche, first live birth, time to recurrence, type of surgery, the type of recurrence (DCIS or invasive cancer), use of adjuvant therapy in the form of radiation or endocrine therapy (Tamoxifen) and current follow-up status. Hematoxylin and eosin stained paraffin sections of the case-control groups were evaluated for size of DCIS and margin status. The grade of DCIS was classified according to the criteria of Page and Lagios [23]; since grading was not uniformly practiced during the early part of the study period, and complete sets of slides were not available on every patient, we elected to review the grade on the TMA core samples for all study subjects. Free margins were defined as a tumor clearance of 2 mm or greater on review.

### Tissue microarray preparation and immunohistochemical analysis

Areas of morphologically representative, non-necrotic sites in the tumor sections were chosen to prepare tissue microarrays (TMA). From each tumor block, two 1.5-mm cores were obtained using tissue arraying instrument (Beecher Instruments; Silver Spring, MD) TMA blocks were prepared using previously described protocol by Kononen et al[24]. Each TMA block consisted of 8–17 cores per block. The coordinates and clinicopathologic data were documented to identify the cores. Sections of 4-μm thickness were used for immunohistochemical analysis of markers with H&E-stained sections as morphological references for each core. Approximately 30% of the DCIS lesions were not represented on the TMA sections either because no DCIS cells were present in a section or complete absence of a core secondary to tissue exhaustion in the block. We attributed this to the nature of many of the samples that contained small and/or scattered areas of DCIS in the donor blocks. Because of this sample loss, there are different samples sizes for each molecular marker.

Molecular markers that have been identified in invasive cancer as having predictive and prognostic value were chosen for our study. These included estrogen receptor alpha (ER-α), progesterone receptor (PR), HER-2/neu, COX-2, PPARγ, cyclin D1, p53, and p21. For all of the markers tested, we used standardized automated immunohistochemical techniques (DAKO Autostainer) along with heat induced antigen retrieval. The clones, dilutions and manufacturers of the primary antibodies used for the study are shown in Table 1.

**Table 1:** Antibodies and scoring system used for analysis

Antibody*	Dilution	Clone	Method of scoring
ER-α	1:300	1D5	> 10% positive cells – positive, < 10%- negative
PR	1:400	PgR636	> 10% positive cells – positive, < 10%- negative
HER-2/neu	1:200	**	Scored as +1, +2 and +3, +3- positive, +1/+2 -negative
COX-2	1:100	CX229	Strong positive, weak positive, negative
PPARγ	1:50	E8	Nuclear, cytoplasmic, nuclear+cytoplasmic, negative
P53	1:200	DO-7	> 10% positive cells – positive, < 10% -negative
P21	1:50	SX118	> 10% positive cells – positive, < 10% -negative
Cyclin D1	1:300	DCS-6	> 10% positive cells – positive, < 10% -negative

*All antibodies were purchased from Dako Cytomation except COX-2 (Cayman Chemicals), PPARγ (Santa Cruz Biotechnologies), Cyclin E (Lab Vision Corporation) and CXCR4 (R&D Systems)

** DAKO polyclonal antibody A0485.

Immunohistochemical scoring of cases was performed by a breast pathologist (LKD) with additional review of equivocal cases by a second pathologist (EL), and final scoring by consensus. All scoring was blinded to patient information, including case-control status. Scoring of each marker was also blinded to the scoring for other markers. Specific scoring systems were used for the various sets of markers studied. Nuclear markers were considered positive when the fraction of stained nuclei was ≥ 10% (ER-α, PR, p53, p21 and cyclin D1), regardless of the intensity of the immunostain. HER-2/neu cores were scored using a standard 0,1+, 2+ and 3+ membrane staining intensity scale; 3+ staining of > 10% cells was required for positivity. COX-2 staining was scored using a system similar to that described for COX-2 evaluation of invasive breast cancer on TMA sections[25]. A three-tiered system was utilized for staining intensity: negative, weak and strong staining. Staining was considered positive if strong cytoplasmic granular staining in > 10% of tumor cells was present.

**Figure 1:**
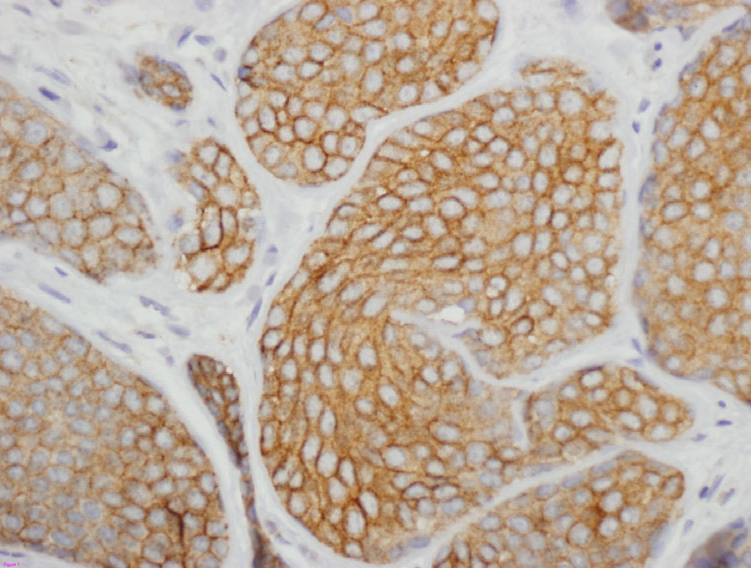
Strong COX-2 positivity on immunohistochemical staining in tissue microarray section of ductal carcinoma in-situ (DCIS) of the breast. Original magnification, ×400.

**Figure 2:**
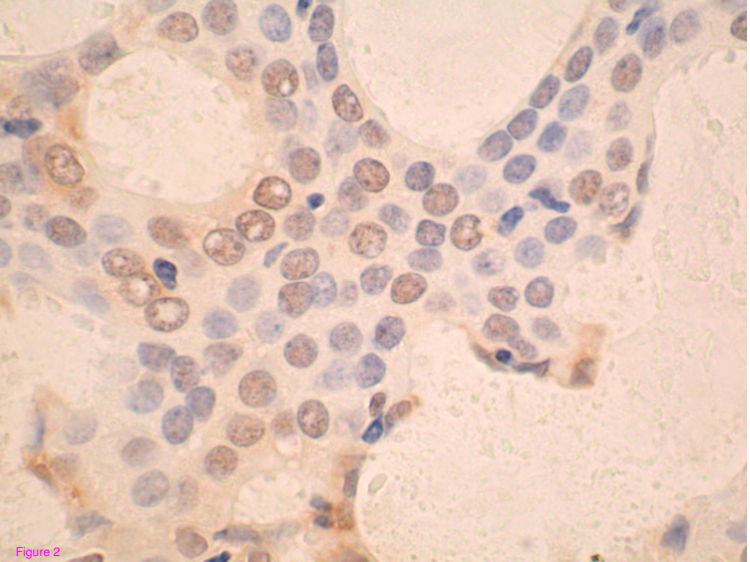
Nuclear positivity for PPAR gamma in tissue microarray section of ductal carcinoma in-situ (DCIS) of the breast. Original magnification, ×400.

PPARγ staining was evaluated using a descriptive scoring system taking into account the possibility of nuclear and cytoplasmic staining patterns as previously described for PPARγ staining in invasive breast carcinoma[26]. We classified cases as: cytoplasmic staining only, combined nuclear and cytoplasmic staining and nuclear staining only. Staining was considered positive if > 10% of tumor cells displayed nuclear staining. Examples of DCIS showing positivity for COX-2 and PPARγ are shown in Figures 1 and 2.

If two cores from the same patient were found to have discordant IHC results, i.e. one core was read as positive and one as negative, or one core was scored as +3 and the second core as +2, the positive result or the higher IHC score was used for the analysis.

### Statistical analysis

All markers were compared between cases and controls. Since there were only eight invasive recurrences, we did not attempt subset analyses of invasive versus non-invasive recurrences. The term "recurrence" is therefore used to encompass both invasive and non-invasive recurrence. Each marker was classified as positive or negative based on the criteria described above and in Table 1. After descriptive statistical analyses, to assess the distribution of each marker, and to check for outliers, a univariate analysis was performed for each marker. Continuous and ordinal measures were compared between cases and controls using the paired t-test or the Wilcoxon signed rank test. Dichotomous measures were compared using the chi-square test. After univariate analysis, multivariate analysis using conditional logistic regression (for matched samples) was used to identify the markers that demonstrated odds ratios that were different from unity, using a threshold p value of 0.1. All p values are two-tailed. The associations between positivity of various markers was explored using Spearman's rank correlation.

## Results

There were a total of 69 women included in the analysis; 31 cases (those women who developed an ipsilateral non-invasive or invasive in-breast recurrence during the study period, 1992–2002) and 38 controls (those women who did not recur). The clinical characteristics of the study population are shown in Table 2. The mean age for the cases was 53.3 years and the mean age for the controls was 53.7 years. Ages ranged from 35 to 80 years. The mean tumor size was 17.7 mm in the cases and 11.9 mm in the controls. This difference was of borderline statistical significance (p = 0.062). The majority of the patients in our study group had DCIS lesions that were classified as histological Grade 2. More Grade 3 tumors were found in the cases in comparison to controls; this difference too, was of borderline statistical significance (p = 0.069). The majority of women (approximately 90% in each group) had free resection margins defined as 2 mm or greater documented on the review of pathologic material performed for this study. Margins of resection were involved with DCIS in three controls and two cases, and were unknown in five controls and one case. All radiotherapy was to the whole breast. The proportion of patients receiving radiation therapy was similar between cases (65%) and controls (61%) and was not associated with recurrence risk (OR 1.2, 95% CI 0.45–3.3) The use of adjuvant tamoxifen therapy was equally infrequent between cases (32%) and controls (32%), and was not associated with a decreased risk of recurrence (OR0.99, 95% CI 0.64–1.51). Many of these patients were treated prior to 2003, when ER testing was not routine clinical practice at our institution. Tamoxifen recommendations were not based on ER status of DCIS prior to this date. When we examined the tamoxifen use based on ER data generated on the TMAs produced for this study, we found no differences in the proportion of ER positive cases and controls who received tamoxifen therapy (7/12 ER positive controls vs. 6/10 ER positive cases).

**Table 2:** Clinical and Pathologic Characteristics of DCIS Lesions in Study Subjects.

	Cases N (%)	Controls N (%)	P value
Number of Subjects	31	38	
Mean age (range)	51 (36–77)	50.5 (35–79)	
Mean size in mm (range)	17.7 (4–73)	11.9 (2–31)	0.06*
DCIS grade:			
Grade 1	5 (16.)	13 (34)	
Grade 2	16 (52.)	18 (47)	
Grade 3	10 (33)	7 (18)	0.07
Free margins (2 mm or greater)	28 (90)	30 (79)	0.72
Invasive recurrence	8 (26)	-	
DCIS recurrence	23 (74)	-	
Tamoxifen Use	10 (32)	12 (32)	0.66
Radiation Therapy	20 (65)	23 (61)	0.71

*T test, two-tailed p value

^~ ^Margin status missing in 1 case and 5 controls

The mean time to recurrence in the cases was 38.5 months (range 6–114 months). Eight cases (26%) experienced an invasive ipsilateral recurrence and 23 cases (74%) had a DCIS recurrence.

Our target retention rate in successive TMA sections was 70% of cores, and was not related to the overall size of the DCIS lesions. There were 18 subjects (4 cases and 14 controls) with target loss of both cores for at least one marker. The mean size of the DCIS in these 18 women was 13.8 mm; the mean tumor size among lesions with target loss was similar to those in which target tissue was retained (17.67 vs. 17.72 mm in cases and 11.4 vs. 11.9 in controls). Thus the loss of DCIS target was not attributable to tumor size (p = 0.7). However, there was a trend towards better retention of DCIS target with increasing grade. Of 18 Grade 1 lesions, target tissue was lost for at least one marker in seven subjects (39%); among Grade 2 lesions the corresponding proportion was 10/34 (29%), and among Grade 3 lesions it was 1/17 (6%) (p = 0.070).

A number of biomarkers were initially evaluated as part of our study. These biomarkers were chosen because of their role in cell cycle regulation, proliferation, differentiation, and apoptosis as well as evidence of prognostic ability in invasive breast cancer. Individually these were found not to be associated positively or negatively with any ipsilateral recurrence (see Table 3), with the exception of COX-2 and PPARγ positivity. The results for COX-2 and PPARγ staining are presented in more detail below.

**Table 3:** Proportion of Cases and Controls with Positive Staining of DCIS Lesions for Markers of Interest

Molecular Markers	Cases N (% positive)	Controls N (% positive)	Total (N)	P value
ER	20 (77%)	22 (79%)	54	0.884
PR	21 (81%)	20 (71%)	54	0.422
HER-2/neu	14 (56%)	18 (64%)	53	0.538
P53	17 (74%)	19 (63%)	53	0.413
Cyclin D1	16 (64%)	16 (59%)	52	0.726
COX-2	18 (67%)	9 (29%)	59	0.006
PPARγ	1 (4%)	7 (27%)	51	0.024
P21	9 (37%)	7 (23%)	54	0.257

### COX-2 Expression in DCIS

COX-2 expression could be evaluated in 59 samples; cores from 10 samples (6 controls and four cases) were lost in the TMAs. Of the 59 women with evaluable COX-2 staining, 45.7% had strong COX-2 expression. (Fig 1) There was a significant difference in the proportion of cases that expressed COX-2 by immunohistochemistry compared to controls. (P = 0.006). Strong cytoplasmic COX-2 staining was found to be associated with ipsilateral breast recurrence after breast-conserving treatment of DCIS. The odds ratio (OR) on univariate analysis was 5.11 (95% CI 1.7–15.5). We did not see a significant association between strong COX-2 expression and histological grade of the lesions, although the majority of Grade 1 lesions were COX-2 negative (9/13, 69%). In the 10 samples where COX-2 expression data were missing due to core loss, the mean size of the DCIS lesions was smaller in the 4 cases (7.25 mm, range 4–15 mm) than in the six controls (12.8 mm, range 2–25 mm).

Although this study lacked the statistical power to examine non-invasive and invasive recurrences separately, it is noteworthy that four of the six cases with invasive recurrences were COX-2 positive. (One of the six was negative and the other case was lost during processing). There was no difference in COX-2 expression between younger women and older women (using an age threshold of 50 years). Although other studies have indicated that HER-2/neu over-expression is associated with DCIS recurrence[7], we did not find an association between HER-2/neu over-expression and ipsilateral DCIS recurrence (p = 0.538). The expression of HER-2/neu and COX-2 was concordant in 29/52 lesions that could be assessed for both proteins (12 lesions were positive for both and 17 lesions were negative for both). Dual negativity for COX-2 and HER-2/neu was significantly associated with control status (17 of 27 controls were dual negative, p = 0.024). Dual positivity for COX-2 and HER-2/neu was not associated with an increase in recurrence risk over COX-2 positivity alone.

### PPARγ Expression in DCIS

PPARγ positivity was evaluable in 51 women (25 cases and 26 controls); nuclear staining was seen by immunohistochemistry in eight of 51 women (15.6%). This includes patients who have nuclear staining alone or nuclear and cytoplasmic staining (Fig 2). Only one of the cases expressed nuclear PPARγ gamma, compared to seven of 26 (27%) of controls. This difference was statistically significant (p = 0.024). Interestingly, expression was limited to grade 2 and 3 DCIS with a highly significant interaction (p < 0.00001) between PPARγ and grade. The odds ratio for recurrence was 0.11 (95% CI 0.012–1.00, p = 0.050) indicating a protective effect of nuclear PPARγ against recurrence. There was no association between age or HER-2/neu status and PPARγ expression. The protective effect of PPARγ positivity was not changed in multivariate modeling with tamoxifen, radiation therapy, tumor size, estrogen receptor status and margin status. Among the group where PPARγ data was missing as a result of processing-related core loss, 12 samples were from controls and 6 were from cases. Similar to the core loss pattern seen with COX-2 staining, the tumor size in case lesions with core loss was similar to that of control lesions with core loss during PPARγ staining (6.8 mm, range 4–20 mm in cases and 8.7 mm, range 2–20 mm in controls).

### Results of combined analysis of Grade, COX-2 and PPARγ expression

We performed a multivariate analysis including the four parameters which were significant at a p value of 0.1 or lower (size, grade COX-2, PPARγ). COX-2 expression continued to be significantly associated with increased risk of any ipsilateral in-breast recurrence, with an OR of 7.89 (95%CI 1.7–36.2). Tumor size remained positively, but not significantly, associated with recurrence risk, with a 5% increase in risk for each mm increase in size (OR 1.05, 95% CI 0.97–1.1). The association of grade and ipsilateral breast recurrence also remains statistically non-significant, although the point estimate remained elevated, with an OR of 1.58 (95% CI 0.5–4.9). In this multivariate model, PPARγ expression remained negatively associated with recurrence, with an OR of 0.17 but this was no longer statistically significant (95% CI 0.02–1.8), (see Table 4).

**Table 4:** DCIS recurrence risk related to histologic grade, COX-2 positivity, and PPARγ positivity.

Univariate Analysis	OR	95% CI	p value
Size	1.05	0.99–1.10	0.081

Grade	1.92	0.95–3.87	0.069
COX-2	5.11	1.7–15.5	0.004
PPARγ	0.11	0.01–1.0	0.05
			
Multivariate Analysis			
Size	1.05	0.97–1.15	0.237
Grade	1.58	0.50–4.93	0.434
COX-2	7.90	1.72–36.23	0.008
PPARγ	0.17	0.06–1.84	0.144

### Associations between markers

Relationships between the markers tested were explored to look for consistency with known correlations, and to identify additional associations which may be of biological interest. These are shown in Table 5. COX-2 expression and PPARγ expression in DCIS had a significant inverse relationship, consistent with their opposite effects on recurrence risk As expected, estrogen and progesterone receptor positivity was highly and significantly correlated (p < .0001), and progesterone receptor expression displayed a significant inverse relation with HER-2/neu positivity. The expected association between ER positivity and Cyclin D1 was also observed [27,28], as was the positive association between ER and p21 positivity. [16]. There were weaker, although significant associations of p53 positivity with ER and cyclin D1 positivity, which are of uncertain significance. Also noted was a strong and significant association between p21 and cyclin D1 positivity, consistent with the suggestion that cyclin D1 expression may be indirectly induced as a result of the accumulation of wild-type p53 through p21 [16].

**Table 5:** Correlations between biomarker positivity for all lesions (case and control)

	PPARγ	COX-2	ER	PR	Cyclin-D	HER2/neu	P53
PPARγ							
COX2	-.310*						
ER	.096	-.035					
PR	-.062	-.0667	.673***				
Cyclin D	.092	-.104	.318**	.497***			
HER-2/neu	-.102	.183	-.164	-.389**	-.089		
P53	.010	.142	.206*	.163	.233*	.194	
P21	-.006	-.036	.256*	.414***	.488***	.123	.391***

Spearman correlations

.* less than 0.05 ** less than 0.005 ***less than .0005

## Discussion

Our results show that strong cytoplasmic COX-2 expression by immunohistochemistry is an independent predictor of increased risk of ipsilateral in-breast recurrence. Furthermore, even though the number of cases was small, COX-2 expression appears to be associated with the development invasive recurrence. The risk of recurrence associated with strong COX-2 positivity was independent of tumor size, margin status, estrogen receptor positivity, use of radiotherapy, and tamoxifen use. This report is corroborated by a recent study, where the COX-2 positivity rate in recurrent DCIS (62%) was very similar to ours (67%) although COX-2 positivity by itself was not a statistically significant predictor of recurrence in that study (p = 0.08)[29]. Other studies have shown an association between COX-2 overexpression and an aggressive phenotype in invasive breast cancer[30,31] and several studies suggest that COX-2 expression is associated with a worse prognosis [32-34]. Together, these findings are exciting for several reasons. COX-2 could potentially be incorporated into the clinic as a prognostic marker for ipsilateral in-breast recurrence, especially invasive recurrence. Excellent antibodies exist for COX-2 staining, it is easily reproducible and scoring is straightforward. Prostaglandin E_2_, the primary product of COX-2, induces inflammation and can act as a mediator in signal transduction pathways that modulate cellular adhesion and cell growth[35,36]. COX-2 inhibitors were being actively investigated in clinical trials for treatment of breast cancer and as chemopreventive agents in high risk women[37], until toxicity concerns interrupted many of these trials. Our results provide added incentive to develop and test new agents to target this pathway, known to be of importance in several epithelial malignancies. We found that roughly half of the ER positive DCIS lesions in our study overexpressed COX-2 protein, the rate limiting enzyme in prostaglandin synthesis. The use of aromatase inhibitors may be particularly useful in these patients given the evidence that aromatase expression is upregulated by prostaglandins, and aromatase mRNA expression is suppressed by COX-2 inhibitors[38].

Several studies have identified younger age as a factor that increases the risk of recurrence after BCT for DCIS[39,40]; we attempted to determine if this was explained by higher COX-2 expression in younger women, but did not find an association between age and COX-2 expression, in agreement with previous studies[31]. We also examined the association between COX-2 expression and HER-2/neu over-expression. In invasive cancers, COX-2 expression is more often found in those patients who are HER-2/neu positive[41], but in a separate study of women with node negative breast cancer, the prognostic value of COX-2 positivity was independent of HER-2/neu amplification[42]. We found a similar pattern in DCIS, in agreement with a recent study[18]; however, we did not note an increased risk of recurrence with co-expression of HER-2/neu and COX-2 over patients who expressed COX-2 alone.

As far as we know this is the first report of PPARγ expression in human DCIS, although one previous study does show a favorable effect of PPARγ positivity on prognosis in women with invasive ductal cancer[26]. PPARγs are members of the nuclear receptor super-family that includes steroid, retinoid and thyroid hormone receptors[43,44]. There are three isoforms: PPARα, PPARδ and PPARγ, which are encoded by different genes and demonstrate organ-specific variation in expression throughout the body. Two forms of PPARγ (PPARγ_1_and PPARγ_2_) have been identified. PPARγ_2 _is expressed only in fat cells; PPARγ_1 _has been found to be expressed in normal breast epithelium and breast cancers[45]. PPARγ as well as the other PPARs hetrodimerize with the retinoid X receptors (RXRs) and then bind to the peroxisome proliferator response elements (PPREs) in the promoter regions of target genes. Ligand binding causes a conformational change in the heterodimer that causes the co-repressor protein to dissociate allowing for activation of gene transcription that is responsible for cell cycle modulation, cellular differentiation, decreased proliferation, and inhibition of angiogenesis[21,22]. Natural low affinity ligands of PPARγ include fatty acids and ecosanoids such as prostaglandin J_2 _(PGJ_2_). Thiazolidinediones, an FDA approved class of anti-diabetic drugs, are highly selective activating ligands for PPARγ at doses used to treat diabetes[46,47].

Our study suggests that the presence of PPARγ in DCIS may be protective against ipsilateral recurrence particularly in Grade 2 and 3 lesions. However, because of the higher rate of Grade 1 core loss during processing, PPARγ positivity was determined in only 9 of the 18 grade 1 DCIS lesions in the study. All nine of these grade 1 DCIS lesions were negative for PPARγ; six of these tumors were in the control group. It is possible therefore (and even likely, given the association of PPARγ expression with Grade 1 in invasive breast cancer[48]) that PPARγ expression in Grade 1 DCIS is more frequent than what we see in our population, but this awaits a larger study, and may require the use of whole sections rather than TMAs, As with COX-2 positivity, there was no association of PPARγ positivity with age or HER-2 status, although it has been reported in one study that PPARγ may inhibit HER-2 pathways in cell culture models[49].

Previous studies have shown that PPARγ ligands were able to cause terminal differentiation of liposarcoma[50]. In breast cancer, numerous cell culture models have shown PPARγ ligand activation induces differentiation and inhibits proliferation[45,51]. Only one study to date has looked at PPARγ ligands in human breast cancer. It was undertaken in a group of women with refractory breast cancer. No response was seen after treatment[46]; however, the authors did not examine tumors for PPARγ expression, and if this is associated with a favorable phenotype, it is possible that these women with refractory advanced disease had largely PPARγ negative tumors.

It is not surprising to find an inverse relationship between expression of COX-2 and PPARγ, given their respective biologic functions. In invasive breast cancer, PPARγ mRNA is significantly decreased compared to normal tissues[52,53] and COX-2 levels have been shown to be upregulated in breast cancer in multiple studies[54,55]. This inverse relationship has been demonstrated previously in invasive carcinoma[52]. To our knowledge this is the first time this relationship has been shown in DCIS. Evidence for a coordinated relationship between COX-2 and PPARγ comes from studies that showing activation of PPARγ suppresses transcriptional activation of COX-2 in cell culture models[41]. Another theory for this inverse relationship has to do with one of the products of constitutive Cyclooxygenase-1, PGJ_2_, a PPARγ ligand, which is downregulated in a paracrine fashion by PGE_2, _the major product of inducible COX-2[56]. We devised a scoring system, combining COX-2 and PPARγ status with size and grade, two important prognostic factors in DCIS.

The limitations of our study include the relatively small size, which was related to the need for tissue from the time of the initial diagnosis, limiting eligibility to patients having their initial surgery at Northwestern Memorial Hospital. Tumor size and grade, both of which have been found to predict recurrence risk in other studies, demonstrated a similar trend in this study, but were of borderline significance, most likely related to the small size of the study. We found that 25% of our patients had grade 3 DCIS, whereas the proportion of grade 3 DCIS in published series ranges from 28% to 50% [57-59] or higher [29]. Since grading of DCIS was not uniformly practiced at NMH over the course of the study, we reviewed grade on all study subjects, based on the TMA core samples. It is possible that the evaluation of DCIS grade on the TMA cores accounted for the high number of grade 2 cases, since heterogeneous distribution of necrosis in the DCIS lesion may not have been captured in the TMA cores, leading to the misclassification of some grade 3 lesions. This may also have reduced our power to detect the effect of grade on recurrence risk, and the high proportion of HER-2/nue positivity among grade 2 lesions may be explained on the same basis. However, we do see the expected inverse relationship between hormone receptor positivity and HER-2/neu positivity [60], which is highly significant for progesterone receptor, which tends to validate our HER-2/neu scoring.

Adjuvant therapy parameters such as tamoxifen and radiotherapy, were also not associated with recurrence, and interactions of these important variables with expression of candidate biomarkers such as COX-2 and PPARγ cannot be meaningfully explored. However, it is not surprising that neither the radiation, nor the tamoxifen benefit seen in large randomized trials is recapitulated in this small case-control study, where the use of radiotherapy (65 vs. 61%) and tamoxifen (32 vs.32%) was equally frequent among cases and controls. Since radiotherapy and tamoxifen exposure were not parameters under study in this case-control design, this balance between cases and controls is an advantage in terms of examining the effects of other parameters. Our rate of invasive recurrence was somewhat lower than reported in large trials [48], not unexpected with a single institution sample where the majority of patients received radiotherapy, and consistent with the proportion of invasive recurrences seen in the radiotherapy arm of NSABP B-18 [61].

Another challenge was the missing data on approximately 30% of tissue cores from the tissue microarrays. We chose to construct tissue microarrays rather than using whole sections because of the greater efficiency of this approach, in order to evaluate multiple biomarkers in this exploratory study. Our success in retaining target tissue through multiple sections of the TMA blocks was approximately 70%, which compares favorably with other reports in the literature regarding target retention in TMAs of pre-invasive lesions [62-64]. The retention rate for pre-invasive lesions of interest in these studies ranged from 52%[64] to 79% [63]; Yang et al noted that target retention for non-invasive targets (DCIS and terminal duct lobular units) remained at about 79% for the first 30 sections, and then declined to 64% at section 40 and 52% at section 100. We noted the same trend, with attrition of target retention as the sectioning progressed through a TMA block. We used a core size of 1.5 mm, and achieved a target retention rate close to that of Yang et. al., who advocates a core size of 2 mm. Target retention was not related to lesion size, in agreement with Yang et. al. [63]

We did not observe an effect on recurrence risk of the additional markers that we tested (ER, PR, HER-2/neu, cyclin D, p53, p21). This is consistent with findings from two other studies of multiple biomarkers and recurrence risk, which included several of the markers that we have examined [58,60]. These authors found no significant impact of ER, PR, or HER-2/neu expression on recurrence risk in multivariate analyses, although Ringberg et. al. did observe a significant relation between a biologic index consisting of seven different markers and risk of DCIS recurrence on women who did not receive breast irradiation. In another study, absence of HER-4 expression was found to be a predictor of recurrence risk [65]. However, our biomarker findings are validated by the observation of many of the expected inter-relations between various biomarkers, as shown in Table 5. Proteins along the ER axis were significantly correlated: ER with PR, cyclin D1 and p21; PR with p21, and inversely with HER2/neu; cyclin D1 with p21. The lack of association of PPARγ with ER or PR is likely related to the small number of PPARγ positive lesions, since such associations have been reported in invasive breast cancers [48]

## Conclusion

These data suggest that overexpression of COX-2 in DCIS lesions is a strong risk factor for local recurrence in the conserved breast; and that PPARg expression may protect against such recurrence. A larger study is needed to validate our findings, which might require a multi-institutional effort. If validated, both COX-2 expression and PPARγ expression would provide additional important prognostic information in DCIS where current tools to predict recurrence are inadequate. Additionally, there is the potential for both agents to be administered together given the cross talk between the two markers. Lastly, given that COX-2 and PPARγ are expressed in both invasive and noninvasive breast cancer, it would be useful to study COX-2 and PPARγ expression in atypical proliferations to see if these biomarkers will improve risk estimation, and in the hopes of identifying molecular targets for chemoprevention.

## Competing interests

The author(s) declare that they have no competing interests.

## Authors' contributions

SK participated in study design, assembled the clinical data set, and wrote the initial draft of the manuscript. DP assisted in the assembly of the pathological materials, and in the performance and interpretation of histochemical stains. LKD and ELW were the pathology referees; LKD supervised the construction of the TMAs and participated in study design. MM contributed to the study design and manuscript revision. SAK conceived of the study, and participated in its design and coordination. All authors read and approved the final manuscript.

## Pre-publication history

The pre-publication history for this paper can be accessed here:


